# A study on the determination of risk factors associated with babesiosis and prevalence of *Babesia* sp., by PCR amplification, in small ruminants from Southern Punjab (Pakistan)

**DOI:** 10.1051/parasite/2011183229

**Published:** 2011-08-15

**Authors:** F. Iqbal, M. Fatima, S. Shahnawaz, M. Naeem, R.S. Shaikh, M. Ali, A.S. Shaikh, M. Aktas, M. Ali

**Affiliations:** 1 Institute of Pure and Applied Biology, Zoology Division, Bahauddin Zakariya University Multan Pakistan; 2 Institute of Biotechnology, Bahauddin Zakariya University Multan Pakistan; 3 Department of Parasitology, Faculty of Veterinary Medicine, University of Firat 23119 Elazig Turkey; 4 Faculty of Veterinary Sciences, Bahauddin Zakariya University Multan Pakistan

**Keywords:** sheep, goats, PCR amplification, *Babesia* sp, ovin, caprin, PCR, amplification, *Babesia* sp

## Abstract

Babesiosis is a parasitic infection due to the multiplication of tick borne parasite, *Babesia* sp., in erythrocytes of host, which includes a wide variety of vertebrates including small ruminants causing decreased livestock output and hence economic losses. The objective of the present study was to establish a PCR based method for the detection of *Babesia* sp. in small ruminant population in Southern Punjab and to determine the risk factors involve in the spread of babesiosis. A total of 107 blood samples were collected from 40 sheep and 67 goats in seven districts of Southern Punjab from randomly selected herds. Data on the characteristics of the animals and the herd were collected through questionnaires. 36 blood samples (34% of total) produced the DNA fragment specific for 18S rRNA gene of *Babesia* sp., by PCR amplification, of which 20 were sheep and 16 were goats. Samples from all seven district contained *Babesia* positive samples and prevalence varied between 18 to 68%. It was observed that male animals (P = 0.009) and young animals under one year of age (P = 0.01) were more prone to the parasite. It was observed that herds consist of more than 15 animals (P = 0.007), composed of mixed species of small ruminants (P = 0.022), associated with dogs (P = 0.003) and dogs having ticks on their bodies (P = 0.011) were among the major risk factors for the spread of babesiosis in small ruminants.

## Abstract (fr)

La babésiose est une infection due à la multiplication du parasite *Babesia* sp., transmis par des tiques, dans les érythrocytes de nombreux hôtes vertébrés dont les petits ruminants, chez qui il est responsable d’importantes pertes de productivité. Les objectifs de l’étude étaient de mettre au point une méthode de détection de *Babesia* sp., reposant sur la PCR, dans des populations de petits ruminants du Penjab du Sud, et de determiner les facteurs de risque impliqués dans la diffusion de la babésiose. 107 échantillons de sang ont été prélevés de façon aléatoire chez 40 ovins et 67 caprins dans des troupeaux de sept districts du Penjab du Sud. Les données concernant les caractéristiques des animaux et des troupeaux ont été collectées à l’aide d’un questionnaire. 36 échantillons de sang (34 % du total) ont révélé la présence du gène spécifique de l’ARNr 18S de *Babesia* sp., par amplification par PCR, chez 20 ovins et 16 caprins. Des échantillons positifs pour *Babesia* ont été trouvés dans les sept districts, avec une fréquence variant de 18 à 68 %. Les animaux de sexe mâle (P = 0,009) et les jeunes de moins d’un an (P = 0,01) étaient les plus touchés par le parasite. Les principaux facteurs de risque de diffusion de la babésiose chez les petits ruminants sont : les troupeaux de plus de 15 têtes (P = 0,007), composés d’un mélange de plusieurs espèces (P = 0,022), associés à la presence de chiens (P = 0,003) et à des chiens porteurs de tiques (P = 0,011).

## Introduction

Babesiosis is a parasitic infection due to the multiplication of *Babesia* sp. in erythrocytes (Levine, 1985; Piesman, 1987). Genus *Babesia* consists of group of intracellular parasites with around hundred species. Clinical signs of disease caused by *Babesia*, babesiosis, are variable including fever, icterus, hemoglubinuria and anemia in the host (Uilenberg, 2001; Yakhchali & Hossein 2006). These tick borne parasites infect a wide variety of vertebrate hosts including small ruminants (sheep and goat) and cattle (Persing & Conrad, 1995). In Pakistan ticks belonging to *Haemophysalis* sp. and *Hyalomma* sp. are involved in the transmission of various piroplasms to small and large ruminants (Rehman *et al.*, 2004).

The economic losses due to babesiosis in sheep and goat production are significant in tropical and subtropical areas (Mehlhorn & Schein, 1984; Bai *et al.*, 2002) and remain an important impediment to meat and milk production because infected animals exhibit high parasitemia and mortality (Caracappa, 1999). Babesiosis in domesticated small ruminants is due to at least three species *Babesia motasi*, *Babesia crassa* and *Babesia ovis* (Friedhoff, 1988). *Babesia ovis* is pathogenic especially in sheep and its case-fatality in susceptible hosts range from 30 to 50 % in field infections (Hashemi-Fesharki, 1997). It leads to significant losses among small ruminants due to its drastic effect on hemobiotic system (Radostits *et al.*, 2000).

The diagnosis of small ruminant piroplasmosis is based on the microscopic examination of Giemsa stained blood smears and clinical symptoms in acute cases. After acute infections, recovered animals frequently sustain sub clinical infections, which are microscopically undetectable (Calder *et al.*, 1996). They can be considered as a source of infection for the potential vector causing natural transmission of the disease. Serological methods are frequently employed in determining sub clinical infections. However, serology for detecting carrier state lack specificity and sensitivity, especially for infection status (Passos *et al.*, 1998). Therefore, DNA amplification methods, which are more sensitive and specific than other conventional methods may facilitate and use a powerful tool for the diagnosis of babesiosis (Aktas *et al.*, 2005, Altay *et al.*, 2005, Nagore *et al.*, 2004).

The aim of the present study was to optimize a specific, reliable and sensitive molecular tool, the polymerase chain reaction (PCR), for the detection of *Babesia* sp. in blood of small ruminants as parasite cannot easily be diagnosed by examination of stained blood film and negative microscopic examination does not exclude the possibility of infection. The objective of this pilot study is to provide baseline data regarding the prevalenece of *Babesia* sp. and risk factors involved in the spread of babesiosis in southern Punjab as to our knowledge, this parasite has never been reported in Pakistan.

## Materials and Methods

**Fig 1.**
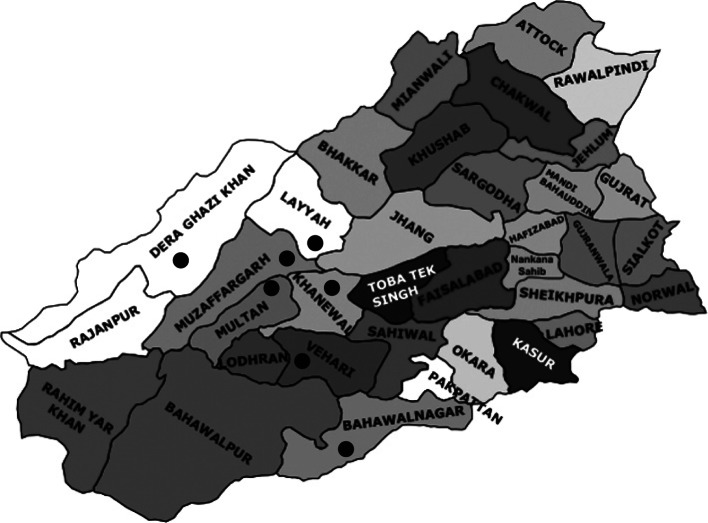
Map of Punjab. Sampling sites in southern Punjab are marked with circles.

Whole blood samples were collected from 107 clinically healthy small ruminants (40 sheep, 67 goats) from randomly selected herds from seven districts (Multan, Muzaffar Garh, Layyah, Dera Ghazi Khan, Khanewal, Vehari and Bahawalnagar) in the important livestock production regions of Southern Punjab (Fig. 1). Blood of 10 % animals from each herd was sampled from jugular vein in Eppendorf tubes and preserved by adding few drops of 0.5 M EDTA. Data on the characteristics of animals (species, gender, age, tick presence or absence, prior treatment for babesiosis) and the herd (location, size, species of animals, dogs associated with the herds, presence of ticks on dogs associated with the herds) was collected through questionnaires completed by the investigators on the spot during sample collection in order to calculate the risk factors involve in the spread of babesiosis. All the experiments were approved by the research and ethic committee of Bahauddin Zakariya University Multan, Pakistan.

Inorganic method of DNA extraction was used following Shaikh *et al*. (2005). The quality of the DNA extract in regard to purity and integrity was assessed with optical density counts at 260/280 nm and submerged gel electrophoresis. A pair of oligonucleotide primers was used to amplify the 146 bp region of 18S rRNA gene of *Babesia* sp. (Nucleotide sequence data reported in this paper are available in GenBank, EMBL and DDBJ databases under accession numbers AY150058, AY534883, AY150061, AY048113) following Theodoropoulos *et al*. (2006).

PCR was performed in 50 μl of a mixture containing about 500 ng of template DNA, 1X PCR buffer, 10 pmol of each primer, 0.2 mM of dNTPs, 1.5 mM MgCl2, 1 % betaine solution and 1 U of Taq DNA polymerase (Vivantis, UK). Remaining volume was adjusted with double distilled water. PCR amplification was obtained after 35 cycles. Each cycle consisted of a denaturing step of 1 min at 95 C, an annealing step of 1 min at 56 C and an extension step of 1 min at 72 C. Distilled water and *Babesia* sp. DNA (previously isolated from naturally infected sheep, GenBank accession no. AY998123) were used in each test as negative and positive controls, respectively. Ten microliters of the amplification products were visualized on 2 % agarose gel stained with ethidium bromide and observed under UV illumination.

For statistical purposes animals were grouped into two age categories: less than 1-year and more than 1-yearold. Herds were divided into two size categories: herds composed of 1-15 and herds with more than 15 animals. Also, herds were divided according to their composition into three categories: herds with sheep only, herds with goat only and herds containing both sheep and goats. The tick burden of sheep, goats and dogs associated with the herds was also scored. Association between the presence (positive and negative blood samples) of *Babesia* and the various parameters, *i.e.* herd location, herd size, species, gender and age of animal, herd composition, presence or absence of ticks on sheep and goats, presence or absence of ticks on dogs associated with the herd was assessed by contingency table analysis using the Fisher’s exact test (for 2 × 2 tables). Statistical package Mini Tab (Version 16) was used for statistical analysis.

## Results and Discussion

Results of PCR amplification revealed that 36 out of 107 sampled sheep and goats were positive for *Babesia* sp. 20 sheep and 16 goat blood samples were parasite positive. The parasite prevalence varied between 18 % (Dera Ghazi Khan district) to 60 % (Layyah district). Sampling sites, total number of samples collected and positive sample along with prevalence details are mentioned in Table I. Table II presents the prevalence of *Babesia* sp. in sheep and goats in relation to the parameters describing the characteristics of the animals while Table III shows the characteristics of herds associated with the incidence of babesiosis.

**Table I.** Sampling sites along with the total number of samples collected (N) from each district. Prevalence of parasite is given in parenthesis.

	*Babesia ovis*		
District	N	Positive	Negative
Dera Ghazi Khan	38	7 (18%)	31 (82%)
Bahawalnagar	5	1 (20%)	4 (80%)
Multan	18	4 (22%)	14 (78%)
Khanewal	3	1 (33%)	2 (67%)
Muzaffar Garh	13	5 (38%)	89 (62%)
Vehari	5	2 (40%)	3 (60%)
Layyah	25	15 (60%)	10 (40%)
Total Animals (Sheep and Goat)	107	36 (34)	71 (66)
Sheep	40	20 (50%)	20 (50%)
Goat	67	16 (24%)	51 (76%)

**Table II.** Association between parasite prevalence in goats and sheep and the studied parameters describing animal characters.

				*Babesia ovis*		
Animal Type		Parameter	No. of samples	Positive	Negative	P-value
Sheep and Goat	Sex	Male	37	20 (54%)	17 (46%)	0.009
		Female	70	16 (23%)	54 (77%)	**
	
	Age	> 1 year	72	17 (24%)	55 (76%)	0.01
		< 1 year	35	19 (61%)	16 (39%)	**
	
	Tick burden	No tick	96	31 (32%)	65 (68%)	0.522
		One or more ticks	11	5 (45%)	6 (55%)	NS

Sheep	Sex	Male	20	15 (75%)	5 (25%)	0.71
		Female	20	5 (25%)	15 (75%)	NS
	
	Age	> 1 year	20	6 (30%)	14 (70%)	0.67
		< 1 year	20	14 (70%)	6 (30%)	NS
	
	Tick burden	No tick	33	18 (55%)	15 (45%)	0.07
		One or more ticks	7	2 (28%)	5 (72%)	NS

Goat	Sex	Male	17	5 (29%)	12 (71%)	0.528
		Female	50	11 (22%)	39 (78%)	NS
	
	Age	> 1 year	52	11 (21%)	41 (79%)	0.326
		< 1 year	15	5 (33%)	10 (67%)	NS
	
	Tick burden	No tick	62	13 (21%)	49 (79%)	0.083
		One or more ticks	5	3 (60%)	2 (40%)	NS

Probability of Fisher Exact test is mentioned for each parameter: NS = non significant (P > 0.05); ** = significant (P < 0.01).

**Table III.** Association between parasite prevalence in sheep and goats and the studied parameters describing animal and herd characters.

			*Babesia ovis*		
	Parameter	No. of samples	Positive	Negative	P-value
Size of herd	1-15	72	17 (24%)	56 (76%)	0.007
	15-30	35	18 (51%)	16 (49%)	**

Herd composition	Goat only	51	14 (27%)	37 (73%)	0.022
	Sheep only	18	4 (22%)	14 (78%)	*
	Sheep and goat together	38	17 (45%)	21 (55%)	

Association of dog with the herd	Dog absent	76	17 (22%)	59 (78%)	0.003
	Dog present	31	18 (58%)	13 (42%)	**

Tick burden on dog	No	76	19 (25%)	57 (75%)	0.011
	Yes	31	17 (55%)	14 (45%)	*

Probability of Fisher Exact test is mention for each parameter except herd composition where ANOVA is applied: NS = non significant (P > 0.05); * = least significant (P < 0.05); ** = significant (P < 0.01).

DNA amplification by PCR is a sensitive and specific tool as compared to other conventional methods (blood smear formation and serological studies) for the diagnosis of babesiosis (Aktas *et al.*, 2005; Altay *et al.*, 2005; Nagore *et al.*, 2004). The small subunit (SSU) 18S rRNA gene is one of the most important markers for PCR based detection of several parasites including *Babesia* sp. as it is part of the ribosomal functional core and is exposed to similar selective forces in all living beings (Criado-Fornelio *et al.*, 2003).

In the present study, among 107 sampled animals 36 (34 %) were positive for *Babesia* sp., which shows the high prevalence of this parasite in southern districts of Punjab. The results indicated that male sheep and goats (54 %) were more infected than females (23 %). This association between gender and parasite prevalence was statistically significant (P = 0.009) (Table II). Our results are contradictory to Theodoropoulos *et al*. (2006) who reported 17 % prevalence of *Babesia ovis* in female sheep and goats in Greece but they did not find any infected male. Our results demonstrated that animals less than one year old (61 %) were more affected with parasite as compared to older animals (Table II) and this association was statistically significant (P = 0.01) indicating that probably the animals develop immunity against parasite with age. Similar finding were reported by Razmi *et al*. (2003).

We also observed an association, although statistically non significant (P = 0.522), between tick burden and parasite prevalence 45 % of the sampled animals having tick present on them were positive for *Babesia* sp. (Table II) confirming the previous findings by Theodoropoulos *et al*. (2006) and. (Atlay *et al.* 2005) that ticks are involved in the spread of babesiosis.

Among the characters of herds, we observed that herds having more than 15 animals were more affected (P = 0.007) with the parasite (51 %) than herds composed of less than 15 animals (Table III). Also herd consisting of both sheep and goat had more *Babesia* sp. positive animals (P = 0.022) than herds containing sheep or goats only (Table III). Our results coincide with the findings of Theodoropoulos *et al*. (2006) indicating that over crowding of animals is a potential risk factor for the spread of babesiosis.

Results indicated that the presence of dog was a primary source of tick transmission (58 %) to small ruminants as the herds with dogs had higher prevalence of parasite (P = 0.003) than those without dogs (Table III). Also herds having dogs with tick burden had 55 % infected animals indicating that the vector, ticks, might have reached the small ruminants through dogs. This association between tick burdens on dog and parasite prevalence was statistically significant (P = 0.011) complementing the finding of Theodoropoulos *et al*. (2006).

Sheep were more susceptible for babesiosis than goats in the present study. The prevalence of *Babesia* sp. in sheep was 50 % as compared to 24 % infected goats (Table I). Friedhoff (1997) had reported that *Babesia* sp. causes diseases exclusively in sheep and rarely in goats. Moreover, males and sheep less than one year old sheep were more affected as compared to female and sheep older than one year but all these associations were statistically non significant (Table II). Our results coincide with the findings of Friedhoff (1997) that younger sheep are more prone to parasites due to developing immune system. Analysis of the data from sampled goats revealed similar findings as in sheep (Table II).

There is hardly any report on the prevalence of babesiosis in ruminants of Pakistan. To our knowledge this is the first report describing the survey on ovine babesiosis in small ruminants through PCR amplification in southern Punjab. High prevalence of *Babesia* sp. indicates that other species of this genus might be existing as well. In the present study, all 107 small ruminants were raised locally indicating that the babesiosis is endemic in this region. A major reason for high prevalence of parasite in southern Punjab could be the poor hygienic conditions and poverty especially in small towns and villages. In many cases, veterinarians are not available for the help and guidance of livestock owners. By generating the public awareness regarding the risk factors, the prevalence of piroplasms can be significantly decreased resulting in better health and output of sheep and goats.
